# Tread carefully when considering vulvar melanoma in a child or adolescent

**DOI:** 10.1002/ski2.233

**Published:** 2023-06-30

**Authors:** Beth Morrel, Suzanne G. M. A. Pasmans, Antien L. Mooyaart, Irene A. M. van der Avoort

**Affiliations:** ^1^ Department of Obstetrics and Gynecology Erasmus MC University Medical Center Rotterdam Rotterdam The Netherlands; ^2^ Department of Dermatology Center of Pediatric Dermatology Sophia Children's Hospital Erasmus MC University Medical Center Rotterdam Rotterdam The Netherlands; ^3^ Department of Pathology Erasmus MC University Medical Center Rotterdam Rotterdam The Netherlands; ^4^ Department of Obstetrics and Gynecology Ikazia Hospital Rotterdam The Netherlands

Dear Editor,

With great interest we read the review article by Sim et al^1^ recently published online in *Skin Health and Disease* on the relation of genital lichen sclerosus (LS) to melanoma. We would like to bring to the attention of the readers of your journal our recent scoping review and population study^2^ on juvenile vulvar melanocytic lesions including melanoma. There are some striking differences between the two reviews, especially regarding the conclusions and recommendations made.

Sim et al included 5 cases of childhood vulvar melanoma with concomitant LS, all in girls 12 years old or younger. We included one additional case report by Friedman et al^3^ of a vulvar melanoma in a 14‐year‐old also in a background of LS. Of the 6 cases, 3 have been challenged by experts.^4^, ^5^

As noted by Sim, vulvar melanoma in adult women is known to be an aggressive tumour, though they did not discuss the fact that all the juvenile cases published had 100% survival. It should be noted that there are no case reports of vulvar melanoma in juveniles without LS.^6^ Population studies on vulvar melanoma do incidentally include juveniles though they do not differentiate for LS.^2^ In addition, the last case of a juvenile with a vulvar melanoma to be reported dates from 2016.^6^ We question if this reflect lower incidence or better diagnostics?

The concept of atypical melanocytic nevi of the genital type, currently often referred to as atypical genital naevi (AGN), was described by Clark et al^7^ and shown to be an entity typically found in young women, as described by Ribé et al.^8^ Sim et al^1^ did not address the subject as to whether the reported vulvar melanomas in a background of LS in juveniles were truly melanomas, or rather nevi with atypical features^4^, ^5^ as found in AGN. Neither did they address the possible influence of the presence of genital LS on the histological features of the melanocytic lesion. As was shown in our population study,^2^ Ki‐67 positivity, a marker for malignancy, was seen to some extent in 92% of the cases of vulvar naevi in juveniles if and only if LS was present, while none were a melanoma. Moreover, our population study showed no vulvar melanomas ever recorded in a juvenile in the Netherlands national cancer registry.

We agree completely with Sim et al^1^ that counselling and follow‐up are important. However, in juveniles any invasive and, thus, potentially mutilating procedures should preferably be avoided. We note that the statistic cited by Sim of childhood malignant melanoma of 0.8–8 per million is of all melanomas in children, the cases of genital malignant melanoma in childhood being at most a small fraction of that number. This does not mean one should stop being vigilant: for a condition so rare, multidisciplinary follow‐up in an expert centre is recommended, for boys, girls and, for that matter, adult men and women alike.

In conclusion, we maintain that the diagnose of vulvar melanoma in a background of LS in childhood or adolescence is contentious, and such a lesion is more likely an AGN or a benign lesion showing atypical features.^2^, ^4^, ^7^ Given the estimated prevalence of VLS in childhood of at least 1:900 and the extreme rarity of vulvar melanoma in a juvenile, if it truly exists at all, the question remains if the occurrence together is simply coincidental. Rightly so, clinicians and pathologists are, in general, warry of under‐diagnosis of any melanoma. But diagnostics surrounding the analysis of a vulvar melanocytic lesion in a juvenile is a doubled‐edged sword: clinicians and pathologists should be aware of the risks of mistaking an AGN for a melanoma. We contend that clinicians should refrain from invasive diagnostics and, can best refer the child to an expert‐centre for clinical follow‐up. These fascinating questions deserve further attention from the professional community in order to avoid unnecessary invasive and possibly mutilating procedures in children and adolescents.

## CONFLICTS OF INTEREST STATEMENT

None to declare.

## AUTHOR CONTRIBUTIONS

**Beth Morrel**: Conceptualization (Lead); Writing – original draft (Lead); Writing – review & editing (Equal). **Suzanne G. M. A. Pasmans**: Writing – original draft (Equal); Writing – review & editing (Equal). **Antien L. Mooyaart**: Writing – original draft (Equal); Writing – review & editing (Equal). **Irene A. M. van der Avoort**: Conceptualization (Lead); Writing – original draft (Lead); Writing – review & editing (Equal).

## FUNDING INFORMATION

This research received no specific grant from any funding agency in the public, commercial, or not‐for‐profit sectors.

## ETHICS STATEMENT

Not applicable.

## Data Availability

There are no research data to be shared.
